# A qualitative study of parent and childcare leadership perspectives on attendance policies at childcare centers

**DOI:** 10.1017/ash.2022.81

**Published:** 2022-05-16

**Authors:** Nicole Poole, Brooke Dorsey-Holliman, Leisha Anderson, Sean O’Leary, Chloe Glaros

## Abstract

**Background:** Attendance policies for common pediatric illnesses vary widely across childcare centers despite nationally published guidelines from the American Academy of Pediatrics. The COVID-19 pandemic has exacerbated this problem, leading to economic loss from parental work absenteeism and excess medicalization of children with common illnesses. We sought to understand barriers to and recommendations for adopting best practices on attendance policies at Early Head Start and Head Start (EHS/HS) childcare centers. **Methods:** We conducted 19 semistructured qualitative interviews: 9 with childcare leadership and 10 with parents from EHS/HS childcare centers across Colorado. Interviews took place between April and December 2021. Interviews were audio-recorded, transcribed, and coded in ATLAS.ti using a priori and emergent coding strategies. Descriptive content analysis was used to identify central themes, which were iteratively revised by 2 authors. **Results:** We derived 7 convergent and 4 divergent themes from leadership and parents addressing attendance decisions. Overlapping themes on barriers to adopting best practices included difficulty assessing symptom severity, limited medical provider understanding of childcare requirements, parent employment pressures, and the impact of the COVID-19 pandemic on exclusion durations. Leadership and parent perspectives differed on resources utilized, understanding of exclusionary symptoms, and role of medical providers in making attendance decisions. Overlapping themes on recommendations for best practices included access to registered nursing, concrete guidance on symptoms, and partnering with health departments. Leadership and parents agree that the COVID-19 pandemic led to increased guideline use in making attendance decisions and increased rates of excluding children from class for minor illness compared to prepandemic times. Both leadership and parents recommended consistency in exclusion practices, but leadership and parents identified medical providers and childcare leadership, respectively, as current sources of inconsistency. Salient findings showed variability in defining a fever by age from both leadership and parents. **Conclusions:** Coordination is needed between childcare centers, medical facilities, and health departments to improve attendance decisions for common pediatric illnesses. Future work should (1) develop concrete symptom guidance for parents with specific exclusion criteria (eg, via a decision aid), (2) assess the utility and feasibility of regular classroom access to registered nursing, and (3) advocate for employee protections to care for sick children at home.

**Funding:** None

**Disclosures:** None

